# *Pectobacterium carotovorum* Subsp. *brasiliense* Causing Soft Rot in Eggplant in Xinjiang, China

**DOI:** 10.3390/microorganisms11112662

**Published:** 2023-10-30

**Authors:** Wei He, Wenfang Luo, Junhui Zhou, Xiafen Zhu, Jianjun Xu

**Affiliations:** 1 Key Laboratory of Integrated Pest Management on Crops in Northwestern Oasis Ministry of Agriculture, Institute of Plant Protection, Xinjiang Academy of Agricultural Sciences, Urumqi 830091, China; hewei8299@163.com (W.H.); lf576263465@163.com (W.L.); junhuiqzhou@163.com (J.Z.); xaifenzhu@163.com (X.Z.); 2 College of Agriculture, Xinjiang Agricultural University, 311 Nongda East Road, Urumqi 830052, China

**Keywords:** pathogenicity, multilocus sequence analysis, first report, host range

## Abstract

An outbreak of stem rot in eggplants was observed in Heshuo County, Xinjiang, during winter 2021–2022 in about 12–35% of the eggplants in the region (about 40 hm^2^). The infected tissues yielded a total of four bacterial strains, which were subsequently subjected to physiological and biochemical assays as well as molecular identification. Based on these analyses, the pathogen was identified as *Pectobacterium carotovorum* subsp. *brasiliense*. The pathogenicity was confirmed through the fulfillment of Koch’s postulates. The host range test confirmed the broad spectrum of species susceptible to infection by the strains. This study represents the first case of infection caused by *P. carotovorum* subsp. *brasiliense* resulting in stem rot in eggplant.

## 1. Introduction

Eggplant (*Solanum melongena* L.) is an important vegetable crop of the sub-tropics and tropics and it is the fifth largest solanaceae crop after tomato, potato, pepper, and tobacco [1]. The eggplant is widely cultivated in India, Bangladesh, Pakistan, China, the Philippines, Egypt, France, Italy, and United States [2]. China is the largest eggplant producer in the world, with a production of 28.4 million tons [3]. However, eggplant diseases cause great yield loss to eggplant production every year. Eggplants are susceptible to virus, bacteria, fungi, and phytoplasma diseases. Several of these diseases result in significant economic losses in the global eggplant production. For instance, verticillium wilt can lead to a yield reduction ranging from 20% to 30% during the late stages of the disease or more under extreme circumstances or total loss in some fields [4]. Bacteria diseases also include some of the most devastating diseases, such as eggplant bacterial wilt [5]. In the south of the Yangtze River in China, the yield of eggplant bacterial wilt is limited to 20–30% in the mild infection season with a low incidence of pathogenic bacteria, and 80–100% in the severe infection season [6].

Bacterial soft rot is a disease that seriously affects plants, especially vegetables and ornamental plants, and can cause soft rot, blackleg, and wilt [7], such as soft rot of potato [8], blackleg of potato [9], tomato bacterial wilt [10]. Soft rot disease can cause serious economic losses to crops, for example, cucumber soft rot disease can cause 20% to 30% yield loss in China [11], and the percentage of infected potato plants has reached 40% to 70%, with 10.5% to 44.7% yield reductions in Serbia [12]. The bacteria in soft rot attack the plant cell walls by secreting pectin lyase, which causes the maceration of the tissue and gives a foul odor [13]. Bacterial soft rot is a disease complex caused by multiple genera of Gram-negative and Gram-positive bacteria. The predominant soft rot pathogens isolated from plants are *Dickeya* and *Pectobacterium*. *Pectobacterium carotovorum* can cause soft rot in *Solanum tuberosum* [14], *Pinellia ternata* [15], *Cucurbita pepo* [16], and *Cucumis sativus* [11]. A number of strains of *P. carotovorum* have recently been reported in Switzerland [17], Korea [18], Venezuela [19], Italy [20], China [11], and Poland [14], including *P. carotovorum* subsp. *carotovorum*, *P. carotovorum*, *P. odoriferum*, and *P. carotovorum* subsp. *brasiliense*, which causes blackleg, leaf blight, and soft rot on potato, paprika, bell pepper, cucumber, tobacco, artichoke, sugarbeet, cabbage, and marrows.

The pathogens of bacterial soft rot include *Erwinia cactica*, *Erwinia carotovora*, and *Erwinia chrysanthemi* [21]. Throughout the course of history, researchers have systematically classified the subspecies of *E. carotovora* into distinct groups based on their characteristic traits, including pathogenicity and host plant origin [22]. To differentiate among subspecies of *E. carotovora*, several researchers have devised robust phenotypic criteria for discriminating among two or more subspecies [23,24,25]. *Erwinia* has been classified into distinct species and subspecies based on the analysis of the 16S rRNA sequences. Subsequently, numerical taxonomy, DNA-DNA hybridization, phylogenetic analysis, and serology have contributed to the identification of five subspecies within *Erwinia*. Furthermore, three subspecies of *P. carotovorum* have been elevated to the rank of species [22].

*P*. *carotovorum* subsp. *brasiliense* was initially identified as the causative agent of blackleg disease in potatoes (*Solanum tuberosum* L.) in Brazil [16], and subsequent studies have revealed its ability to also cause soft rot in *Capsicum annum* L., *Ornithogalum* spp., and *Daucus carota* subsp. *sativus* [26]. Strains of this taxon have been isolated from various countries including the USA, Canada, and Germany [27]. Notably, approximately 20% of the *P. carotovorum* strains collected in Syria were identified as *P. carotovorum* subsp. *brasiliense* [28]. Glasner et al. (2008) demonstrated that *P. carotovorum* subsp. *brasiliense* shares a conserved core genome of approximately 3–9 Mb with both *P. carotovorum* subsp. *carotovorum* (WPP14) and *P. atrosepticum* (strain SCRI 1043) [29]. However, a subset of genes constituting 13% of the chromosome in strain *P. carotovorum* subsp. *brasiliense* 212 ^T^ were found to be unique to this subspecies and not present in either *P. atrosepticum* or *P. carotovorum* subsp. *carotovorum*. Glasner et al. (2008) further proposed that *P. carotovorum* subsp. *brasiliene* should be considered as a separate species based on its unique genomic organization, and this observation was confirmed by amplified fragment length polymorphism (AFLP) analyses [29].

The extensive diversity of the biochemical and genetic characteristics among strains of *P. carotovorum* within the same subspecies presents challenges in accurately identifying this pathogen through physiological, biochemical, and 16S rRNA sequence analysis. Given that *acnA*, *gapA*, *icdA*, *mdh*, *mtlD*, *pgi*, and *proA* genes are commonly found in most enterobacteria, and exhibit sufficient sequence diversity despite not being clustered in the genome [30], serve as useful markers to distinguish *P. carotovorum* strains.

The aim of this study was to isolate and identify the bacteria associated with a recent outbreak of eggplant soft rot, to establish a scientific basis for disease prevention and control strategies.

## 2. Materials and Methods

### 2.1. Sample Collection and Strain Isolation

During winter 2021–2022, severe stem rot in eggplants was observed in the main production regions in Heshuo County, Xinjiang, China (42°55′22″ N, 93°8′25″ E), with disease incidence varying from 12% to 35% in an affected area of approximately 40 hectares.

A total of 15 eggplant stem rot samples were collected from 10 greenhouses in Quhui Town, Hesuo County, Xinjiang, China. To isolate pathogens from infected eggplant stems, diseased tissues were surface-sterilized by immersing them in 75% ethanol for 30 s, followed by transfer to a 3% sodium hypochlorite (effective chlorine) solution for 2 min. Subsequently, the tissues were rinsed three times with sterile distilled water and ground using a rod in a mortar containing 2 mL of sterile distilled water. The ground tissue was allowed to soak for 5 min, following which a liquid suspension of 10 μL was streaked onto Luria–Bertani (LB) agar. Ten isolates with the same colony morphology were obtained from soft rot symptoms, and four strains (ESRB-1, ESRB-2, ESRB-3, ESRB-4) were randomly selected for further study.

### 2.2. Biochemical Characterization

Four randomly selected strains were grown on LB medium. Carbon source utilization was tested by using the Microlog system (Biolog Inc., Hayward, CA, USA). The optical density of the suspension was adjusted as recommended by the manufacturer. Furthermore, in accordance with previous reports, all strains were characterized using standard tests commonly employed for the identification of pectolytic *Pectobacterium* spp. [26].

### 2.3. 16S rRNA Gene Sequence Analysis

The 16S rRNA gene sequences of four isolated strains were amplified using the universal primers 27mF (5′-AGAGTTTGATCMTGGCTCAG-3′) and 1492mR′-GGYTACCTTGTTACGACTT-3′). Total DNA was extracted using PH0210 Bacterial Genome DNA Extraction Kit (31516KC4, AXYGEN, Union City, CA, USA), following the manufacturer’s instructions. PCR amplification was performed on a DL9700 PCR instrument (Donglin, Changsheng, China): 94 °C for 3 min, 25 cycles of denaturation at 94 °C for 30 s, annealing at 54 °C for 30 s, and extension at 72 °C for 1 min 30 s, with final extension at 72 °C for 10 min. PCR products were purified with an NEP025-1Recovery Kit (Dingguo, Beijing, China) and sequencing was performed using a 3730XL sequencer (625-0020, ABI, Los Angeles, CA, USA). The sequences were subsequently subjected to analysis using the BLAST program. Phylogenetic analysis was conducted employing MEGA5 with the maximum likelihood method [31]. The tree is drawn to scale, with branch lengths in the same units as those of the evolutionary distances used to infer the phylogenetic tree.

### 2.4. Multilocus Sequence Analysis

Eight housekeeping genes (Table 1) were used in the PCR amplification [28]. Sequencing was carried out by Roning Biotechnology Co., LTD (Chengdu, China), and sequences were submitted to GenBank under accession numbers OR493415 to OR493418 and OR512996 to OR513023 (Table S1). The housekeeping gene sequences were aligned using CLUSTAL-W in MEGA [31], version 5.0. Genetic analysis was performed on the complete nucleotide sites of each partial gene sequence: *mtlD* (390 bp), *acnA* (300 bp), *icdA* (520 bp), *mdh* (460 bp), *pgi* (520 bp), *gapA* (450 bp), *proA* (630 bp), and *rpoS* (880 bp). An informative maximum likelihood method based on the Tamura–Nei model was implemented in MEGA5, accompanied by a bootstrapping test consisting 1000 replications [32].

### 2.5. Pathogenicity and Host Range Test

To fulfil Koch’s postulates, eggplant plants were surface-sterilized with 1% NaOCl and inoculated with bacterial suspension of 1 × 10^8^ CFU/mL obtained from LB cultures by acupuncture method. The eggplant variety is Kuai yuanqie. Each stem was stabbed at two points, with a distance between of 1 cm. Inoculated plants were placed in plastic boxes with 100% relative humidity at room temperature for 48 h. Then, the inoculated eggplant plants were transferred to an incubator maintained at 17 °C (night) and 28 °C (day) and observed for the presence of soft rot symptoms every day. Sterilized distilled water was used for negative control inoculations.

The host ranges were assessed using 10 crops, encompassing two species from the Cucurbitaceae family (cucumber and zucchini), four species from the Brassicaceae family (Chinese cabbage, carrot, radish, and cabbage), as well as four species from the Solanaceae family (pepper, tomato, eggplant, and potato). All fruit-inoculated crops were purchased from local markets. Paprika fruit was inoculated with acupuncture. The hole of approximately 1 cm in length, width, and depth on the surface of the fruits of the other 9 crops was cut by a sterile scalpel, and 20 μL bacterial suspension (10^8^ CFU/mL) was inoculated into the holes and covered with the cut tubers. Three to five holes were cut into each fruit, and the distance between any two holes was at least 0.5 cm. Inoculated fruits were covered with soaked absorbent cotton and wrapped with plastic wrap at room temperature for 24 h. Then, the inoculated fruits were transferred to an incubator maintained at room temperature and observed for the presence of soft rot symptoms every day. Sterilized distilled water was used for negative control inoculations. The inoculation experiment was repeated three times, and each repeat included three fruits.

## 3. Results

### 3.1. Symptoms of Infected Eggplant Plants and Bacterial Isolation

**Figure 1:**
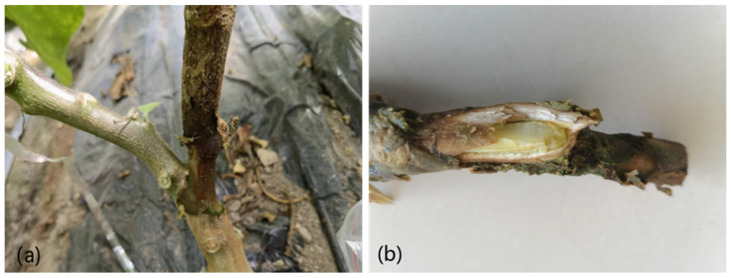
Symptoms of eggplant soft rot disease caused by *P. carotovorum* subsp. *brasiliense*: (**a**), stem rot; (**b**), vascular bundle of stem rot.

The surveys of eggplant soft rot disease were conducted during winter 2021–2022 in Heshuo County, Xinjiang, China. The prevalence of this disease was observed in over 85% of the 50 surveyed greenhouses. The disease mainly affects the stem of the eggplant, which initially presents as a watery surface of infected tissue and water soaking in the infected stems, and later causes stem shrinkage, epidermal decay, necrosis in the vascular tissue, and a foul smell (Figure 1a,b). Four strains (ESRB-1, ESRB-2, ESRB-3, ESRB-4) were isolated from the diseased lesions on the eggplant stems.

### 3.2. Pathogenicity Assay

**Figure 2:**
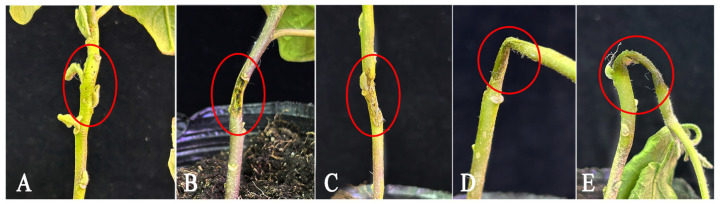
Symptoms of soft rot in eggplant plants were observed 48 h after inoculation with *P. carotovorum* subsp. *brasiliense* strain. (**A**–**E**) were the symptoms at the inoculation site after inoculating with sterile water, ESRB-1, ESRB-2, ESRB-3, and ESRB-4, respectively. In the red circle are inoculation sites.

The four strains were inoculated in the eggplant stem again, and the inoculation site of the eggplant stem constricted, decayed, and released an odour after 48 h (Figure 2). These symptoms were observed on the experimentally inoculated stem which was consistent with the symptoms of the eggplant stem in the field, and the same strains were isolated from the infected site of the eggplant stem.

### 3.3. 16S rRNA Gene Analysis

**Figure 3:**
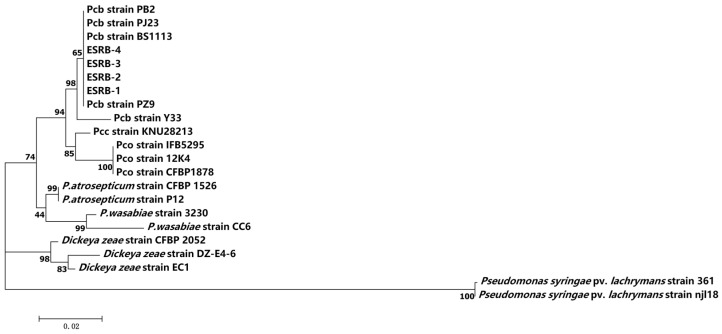
Phylogenetic tree constructed based on 16S rRNA sequence analysis. The numbers above branches indicate bootstrap value, with support derived from maximum likelihood analyses of 1000 bootstrap replications.

The amplification of the 16S rRNA gene resulted in the generation of a 1400 bp sequence for ESRB-1, ESRB-2, ESRB-3, and ESRB-4 (GenBank Accession Nos. OR066157.1, OR066158.1, OR066159.1, and OR066160.1, respectively). This step was pivotal in advancing the comprehension of the phylogenetic relationship among these strains. Upon conducting a phylogenetic analysis based on the 16S rRNA gene, the 22 strains could be categorized into four levels of similarity (Figure 3). The four strains isolated from the eggplants exhibited an identical match of 100% with *P. carotovorum* subsp. *brasiliense* (Pcb). This discovery implies a close genetic association between these isolates and subspecies, potentially warranting their consideration as part of the same species group. Furthermore, these eggplant-derived strains formed a distinct cluster alongside other *P. carotovorum* subsp. *brasieliense* strains as well as a partial strain of *Pseudomonas carotovorum* subsp. *carotovorum* and *Pseudomonas carotovorum* subsp. *odoriferum*.

### 3.4. Multilocus Sequence Analysis

**Figure 4:**
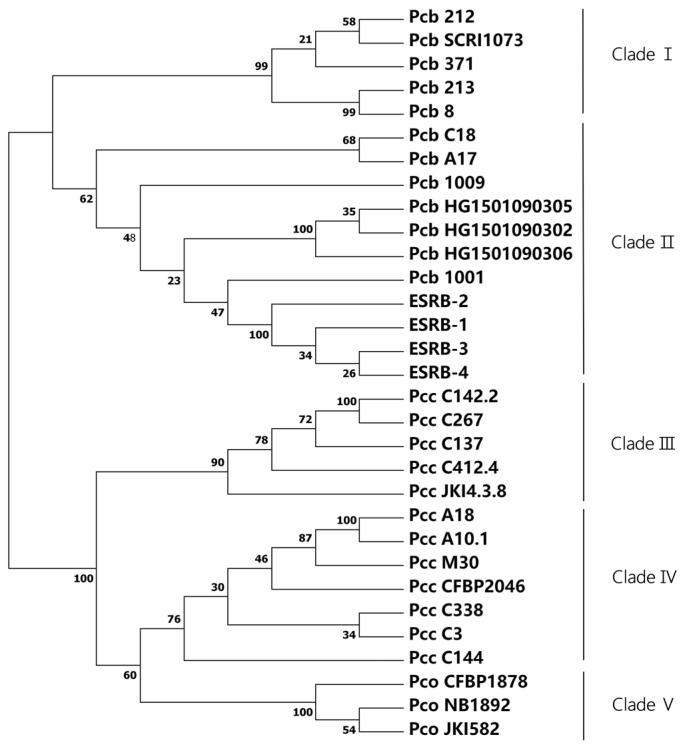
Phylogenetic tree constructed based on sequence analysis of 8 housekeeping genes by the maximum likelihood method.

The taxonomic status of the four strains isolated from the eggplant stems was further determined by concatenating them with 27 related taxa and employing a multilocus sequence analysis. (Figure 4). The results showed that the 31 strains were divided into five clades, of which the *P. carotovorum* subsp. *brasiliense* strains were grouped into clades I and II, *P. carotovorum* subsp. *carotovorum* was grouped into clades III and IV, and *P. carotovorum* subsp. *odoriferum* belonged to clades V, which was consistent with a previous study [11]. The four strains (ESRB-1, ESRB-2, ESRB-3, and ESRB-4) were unequivocally assigned to clade II. These findings provide conclusive evidence that the four selected strains obtained from the eggplant stems from China are classified as *P. carotovorum* subsp. *brasiliense*.

### 3.5. Results of Physiological and Biochemical Analyses

The physiological and biochemical characteristics of four strains were documented in Table 2. The results showed that the four strains isolated from the eggplant stems grow well within 24 h at 37 °C, showed a tolerance of 5% NaCl, and formed pits on CVP media. The four strains were Gram-negative facultative anaerobes. In a biochemical test, the four strains tested positive for acid production from glucose and maltose, reducing substances from sucrose, utilizing acetic acid, cellobiose, D-arabitol, D,l-lactic acid, D-melibiose, D-sorbitol, succinamic acid, thymidine, and uridine, and all strains were negative for 2-deoxyadenosine, D-glucosaminic acid, D-glucuronic acid, glucose-1-phosphate, inosine, L-glutamic acid, maltose, tween-40, and tween-80. These characteristics are consistent with the published data for *P. carotovorum* subsp. *brasiliense* [34].

### 3.6. Host Range Test

**Figure 5:**
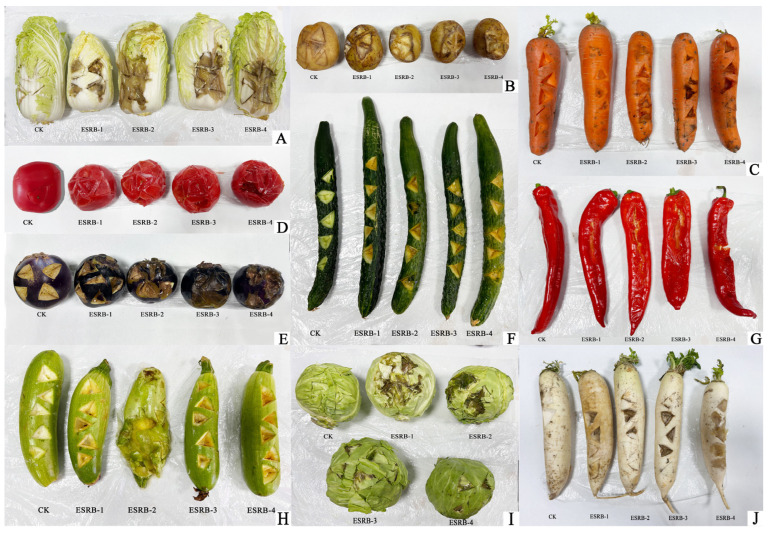
Host range test was conducted for *P. carotovorum* subsp. *brasiliense*, wherein fruits were inoculated with bacterial cells at a concentration of 10^8^ CFU ml^−1^. (**A**), Chinese cabbage; (**B**), potato spinach; (**C**), carrot; (**D**), tomato; (**E**), eggplant; (**F**), cucumber; (**G**), pepper; (**H**), zucchini; (**I**), cabbage; (**J**), white radish.

The comprehensive host range test results revealed that the four strains (ESRB-1, ESRB-2, ESRB-3, and ESRB-4) isolated from the eggplant stems possess the potential to elicit soft rot syndrome in fruits belonging to diverse plant families (Figure 5). The pathogenicity of these strains was observed to be particularly potent as they caused soft rot symptoms in the aforementioned crops within a mere 48 h of initial infection. This finding not only emphasizes the wide-reaching impact of these but also underscores the necessity for stringent measures to mitigate their potential threats to crop production and agricultural sustainability.

## 4. Discussion

Soft rot disease can result in significant economic losses for both monocotyledonous and dicotyledonous plants. *Dickeya* and *Pectobacterium* are extensively studied bacterial pathogens associated with soft rot [27]. *Pectobacterium* species have been reported as pathogens affecting plants from 16 dicot plant families across 11 orders, as well as plants from 11 monocot families distributed in 6 orders [27]. *P. carotovorum* subsp. *brasiliense* causing soft rot in cucumber [11], paprika [18], bell pepper [19], artichoke [20], and potato [21] have been reported. The pathogens that have been reported to cause eggplant soft rot include *Choanephora cucurbitarum* [35], *Pectobacterium wasabiae* [36], *Gilbertella persicaria* [37], *Phytophthora nicotianae* [38], *Erwinia chrysanthemi* [39], and *P. carotovorum* subsp. *carotovorum* [40]. Based on a comprehensive analysis of the physiological, biochemical, and molecular characteristics, *P. carotovorum* subsp. *brasiliense* has been conclusively identified as the primary causative agent responsible for soft rot diseases on eggplant. This study presents a comprehensive investigation into the pathogenicity and host range of the pathogen, establishing a solid groundwork for the development of efficacious control strategies.

The results of the pathogenicity test demonstrate that the strain isolated from the eggplant stem was capable of inducing stem rot, which exhibited symptoms similar to those observed in the field. Moreover, strains re-isolated from the diseased sites displayed identical characteristics to their original counterparts. The bacteria isolated from the stem of the eggplants were identified as *P. carotovorum* subsp. *brasiliense* based on their physiological and biochemical characteristics, 16S rRNA sequencing, and multilocus sequence analysis. This study is the first report worldwide that *P. carotovorum* subsp. *brasiliense* cause soft rot on eggplant stems. This pathogenic bacteria infects the stems of eggplant plants through natural openings or wounds, causing a soft rot that eventually leads to the death of the plant. This disease has the potential to cause significant yield losses, posing a serious threat to eggplant farmers worldwide.

The four strains isolated from the stem of eggplants in Hesocho County, Xinjiang, China, was clearly distinguished from *Pseudomonas syringae* pv. *lachrymans* by the 16S rRNA sequencing method, but it was difficult to distinguish them from the strain *P*. *carotovorum*. It is difficult to distinguish *Pectobacterium* subspecies by 16S rRNA gene sequencing, which is consistent with previous studies [26]. To further distinguish *Pectobacterium* subspecies, multi-locus sequence analysis was used to analyze the bacterial isolates. The results of the multi-gene analysis showed that the 31 strains were divided into five clades, among which, *P. carotovorum* subsp. *brasiliense* was in clades I and II, clades III and IV included *P. carotovorum* subsp. *carotovorum* strains, *P. carotovorum* subsp. *odoriferum* belonged to clade V, and our isolated strains belonged to clade II, which is consistent with the results of previous studies [11].

The most frequently isolated pathogens from plant soft rot samples are *Pectobacterium* spp. And *Dickey* spp., and this suggests that they are the predominant pathogens associated with this disease [27]. *Pectobacterium* spp. and *Dickeya* spp. were the most common pathogen of soft rot in nature. *Pectobacterium* spp. and *Dickey* spp. exhibits an exceptional host range, showcasing remarkable adaptability. Unlike most plant pathogens that are restricted to infecting specific plant groups, *Pectobacterium* spp. and *Dickey* spp. possess the capability to induce diseases in a diverse array of hosts. This extensive host range renders *Pectobacterium* spp. and *Dickey* spp. a formidable pathogen for farmers and agricultural professionals due to its potential for rapid transmission across different plant species, posing significant threats to entire crop systems.

*P*. *carotovorum* subsp. *brasiliense*, an indigenous bacterium from Brazil [41], possesses the potential to induce rapid and severe pathogenicity in plants, resulting in swift tissue decay and occasional plant mortality. The results of this study clearly demonstrate the potent pathogenic bacterium *P. carotovorum* subsp. *brasiliense*, as evidenced by the rapid onset of stem and fruit rot, as well as necrosis, within a mere 48 h period post-inoculation. This bacterium, known to be highly virulent, has been found to inflict significant damage on a wide range of crops including potato [12], tomato [42], and pepper [19], among others. The expeditious manifestation and severity of these observed symptoms underscore the criticality of implementing effective preventive measures such as stringent sanitation practices and hygiene protocols to minimize infection risks and subsequent crop losses. *P*. *carotovorum* subsp. *brasiliense* is renowned for its broad host range encompassing essential crops such as cucumbers [11], paprika [18], kale [41], zucchini [14], cabbage [14], tomatoes [42], and radishes [43]; however, no instances of eggplant soft rot caused by *P. carotovorum* subsp. *brasiliense* have been reported. The results of the host range determination showed *P*. *carotovorum* subsp. *brasiliens* also infect the fruits of 10 crops belonging to four families, including Cruciferae (cabbage and radish), Solanaceae (potato, eggplant, tomato, and pepper), Umbelliferaceae (carrot), and Cucurbitaceae (zucchini and cucumber). This was consistent with the previous reports that *P. carotovorum* subsp. *brasiliense* could induce disease in some crops [9,11,14,19,42].

## Figures and Tables

**Table 1:** Primers sequence for 8 housekeeping genes.

Gene	Primer Name	Primer Sequence	Reference
*acnA*	acnA3F	CMAGRGTRTTRATGCARGAYTTTAC	Ma et al., 2007 [27]
	acnA3R	GATCATGGTGGTRTGSGARTCVGT	
*gapA*	gapA326F	ATCTTCCTGACCGACGAAACTGC	Ma et al., 2007 [27]
	gapA845R	ACGTCATCTTCGGTGTAACCCAG	
*icdA*	icdA400F	GGTGGTATCCGTTCTCTGAACG	Ma et al., 2007 [27]
	icdA977R	TAGTCGCCGTTCAGGTTCATACA	
*mdh*	mdh86F	CCCAGCTTCCTTCAGGTTCAGA	Ma et al., 2007 [27]
	mdh628R	CTGCATTCTGAATACGTTTGGTCA	
*mtlD*	mtlD146F	GGCCGGTAATATCGGCCGTGG	Ma et al., 2007 [27]
	mtlD650R	CATTCGCTGAAGGTTTCCACCGT	
*pgi*	pgi815F	TGGGTCGGCGGCCGTTACTC	Ma et al., 2007 [27]
	pgi1396R	TGCCTTCGAATACTTTGAACGGC	
*proA*	proAF1	CGGYAATGCGGTGATTCTGCG	Ma et al., 2007 [27]
	proAR1	GGGTACTGACCGCCACTTC	
*rpoS*	rpoS1	ATGAGCCAAAGTACGCTGAA	Waleron et al., 2002 [33]
	rpoS2	ACCTGAATCTGACGAACACG	

**Table 2:** Phenotypic characteristics of the strains isolated from eggplant stem.

Characteristic	ESRB-1	ESRB-2	ESRB-3	ESRB-4
Phosphatase	−	−	−	−
Acid from α-methyl glucoside	+	+	+	+
Tolerance of 5% NaCl	+	+	+	+
Growth at CVP medium	+	+	+	+
Growth at 37 °C	+	+	+	+
Acid production from glucose	+	+	+	+
Reducing substances from sucrose	+	+	+	+
Acid production from maltose	+	+	+	+
Utilization of acetic acid	+	+	+	+
Cellobiose	+	+	+	+
D-arabitol	+	+	+	+
2-Deoxyadenosine	−	−	−	−
D-glucosaminic acid	−	−	−	−
D-glucuronic acid	−	−	−	−
D,l-lactic acid	+	+	+	+
D-melibiose	+	+	+	+
D-sorbitol	+	+	+	+
Glucose-1-phosphate	−	−	−	−
Inosine	−	−	−	−
L-glutamic acid	−	−	−	−
Maltose	−	−	−	−
Succinamic acid	+	+	+	+
Thymidine	+	+	+	+
Tween-40	−	−	−	−
Tween-80	−	−	−	−
Uridine	+	+	+	+

## Data Availability

All the data related to this project are presented here.

## Supplementary Materials

The following supporting information can be downloaded at: https://www.mdpi.com/article/10.3390/microorganisms11112662/s1, Table S1: GenBank accession numbers for eight house-keeping gene of four strains obtained from eggplant stem and reference *Pectobacterium* spp. strains.
