# Supplementary material for: Pectobacterium carotovorum Subsp. brasiliense Causing Soft Rot in Eggplant in Xinjiang, China
Source: Microorganisms. 2023 Oct 30;11(11):2662. doi: 10.3390/microorganisms11112662 (PMC10673395; doi:10.3390/microorganisms11112662)
Supplement: Supplementary file 1 [file microorganisms-11-02662-s001.zip › microorganisms-2651113-supplementary.pdf]

Table S1. GenBank accession numbers for eight house-keeping gene of four strains obtained from eggplant stem and reference *Pectobacterium* spp. strains.

Strain	*acnA*	*gabA*	*icdA*	*mdh*	*mtlD*	*pgi*	*proA*	*rpoS*
8	JF926768.1	JF926778.1	JF926788.1	JF926798.1	JF926808.1	JF926818.1	JF926828.1	JF926838.1
213	JF926771.1	JF926781.1	JF926791.1	JF926801.1	JF926811.1	JF926821.1	JF926831.1	JF926841.1
371	JF926770.1	JF926780.1	JF926790.1	JF926800.1	JF926810.1	JF926820.1	JF926830.1	JF926840.1
212^{T}	JF926769.1	JF926779.1	JF926789.1	JF926799.1	JF926809.1	JF926819.1	JF926829.1	JF926839.1
SCRI1073	HM156787	HM156848	HM156910	HM156971	HM157033	HM157090	HM157157	HM157201
1001	JF926767.1	JF926777.1	JF926787.1	JF926797.1	JF926807.1	JF926817.1	JF926827.1	JF926837.1
1009	JF926766.1	JF926776.1	JF926786.1	JF926796.1	JF926806.1	JF926816.1	JF926826.1	JF926836.1
A17	HM156764	HM156824	HM156885	HM156946	HM157008	HM157070	HM157132	HM157194
C18	HM156768	HM156828	HM156889	HM156950	HM157012	HM157074	HM157136	HM157198
JKI4.3.8	HM156790	HM156849	HM156911	HM156972	HM157036	HM157092	HM157158	HM157204
C412.4	HM156784	HM156843	HM156905	HM156966	HM157028	HM157091	HM157152	HM157227
C137	HM156769	HM156829	HM156890	HM156951	HM157013	HM157075	HM157137	HM157210
C142.2	HM156773	HM156833	HM156894	HM156955	HM157017	HM157079	HM157141	HM157214
C267	HM156777	HM156836	HM156898	HM156959	HM157021	HM157083	HM157145	HM157218
C3	HM156767	HM156827	HM156888	HM156949	HM157011	HM157073	HM157135	HM157197
C338	HM156780	HM156839	HM156901	HM156962	HM157024	HM157086	HM157148	HM157221
A10.1	HM156761	HM156821	HM156882	HM156943	HM157005	HM157067	HM157129	HM157191
A18	HM156765	HM156825	HM156886	HM156947	HM157009	HM157071	HM157133	HM157195
M30	HM156785	HM156844	HM156906	HM156967	HM157029	HM157099	HM157153	HM157199
C144	HM156775	HM156835	HM156896	HM156957	HM157019	HM157081	HM157143	HM157216
$CFBP2046^{T}$	JF926762.1	JF926772.1	JF926782.1	JF926792.1	JF926802.1	JF926812.1	JF926822.1	JF926832.1
SCRI2	HM156788	HM156846	HM156908	HM156969	HM157031	HM157104	HM157155	HM157202
$CFBP1878^{T}$	JF926763.1	JF926773.1	JF926783.1	JF926793.1	JF926803.1	JF926813.1	JF926823.1	JF926833.1
JKI582	HM156799	HM156857	HM156919	HM156980	HM157042	HM157102	HM157165	HM157229
NB1892	HM156798	HM156858	HM156920	HM156981	HM157043	HM157103	HM157166	HM157230
ESRB-1	OR493418	OR512996	OR513000	OR5130004	OR5130008	OR513012	OR513016	OR513020
ESRB-2	OR493417	OR512997	OR513001	OR513005	OR513009	OR513013	OR513017	OR513021
ESRB-3	OR493416	OR512998	OR513002	OR513006	OR513010	OR513014	OR513018	OR513022
ESRB-4	OR493415	OR512999	OR513003	OR513007	OR513011	OR513015	OR513019	OR513023
